# Cardiac rehabilitation in Austria

**DOI:** 10.1007/s10354-017-0607-x

**Published:** 2017-11-03

**Authors:** Josef Niebauer

**Affiliations:** Chair of the Institute of Sports Medicine, Prevention and Rehabilitation and Research Institute of Molecular Sports Medicine and Rehabilitation, Paracelsus Medical University Salzburg, Institute of Sports Medicine of the State of Salzburg, Sports Medicine of the Olympic Center Salzburg-Rif, Lindhofstr. 20, 5020 Salzburg, Austria

**Keywords:** Exercise training, Exercise capacity, Quality of life, Outpatient, Inpatient

## Abstract

Cardiac rehabilitation is a class 1 level A indication for the treatment of cardiac diseases. In the following text, details and particularities of the Austrian system will be explained and discussed.

Cardiac rehabilitation (CR) is a class 1 level A indication for the treatment of cardiovascular diseases and has thus received the highest classification possible from major professional societies [1, 2]. This has also been recognized in Austria and guidelines for outpatient cardiac rehabilitation have been written [3]. Indeed, there is reduced morbidity and mortality in patients who receive outpatient CR as opposed to those who do not [4–6]. For inpatient CR there is a paucity of such data, and even though it can be assumed to result in comparable effects, this remains to be shown. In Austria, physicians can request CR (Table 1) for their patients provided their diagnoses are on the list of qualifying indications (Table 2). Most often CR is sought for by hospital physicians after a cardiac event. However, in case this chance has been missed or patients were initially reluctant to opt for CR, a patient’s individual physician can also submit the request.

**Table 1:** Phases of cardiac rehabilitation

*Phase I*
Early in-hospital mobilization after an acute event
*Phase II*
For many patients, outpatient cardiac rehabilitation (phase II: 4–6 weeks) is a suitable and sometimes preferable alternative to inpatient rehabilitation
*Phase III*
Following phase II in- or outpatient rehabilitation, phase III outpatient rehabilitation is offered in order to assure sustainability of the results achieved during phase II rehabilitation
*Phase IV*
Lifelong secondary prevention lies in the responsibility of every patient (heart groups, sports clubs, home training, etc.)

**Table 2:** Indications and contraindications for outpatient cardiac rehabilitation [3]

*Indications*
Acute coronary syndrome (STEMI and NSTEMI)
Aortocoronary bypass surgery
Other surgeries of the heart and the big vessels
Heart and lung transplantation
Chronic heart failure (NYHA stage II, III)
PCI
Stable coronary heart disease
Pulmonary hypertension
Peripheral artery occlusive disease (claudicatio intermittens)
Prevention in motivated high risk patients (SCORE: 10-year risk of cardiovascular death of >5%; PROCAM: coronary event of >20%)
Electrophysiological intervention
Implantation of a cardiac pacemaker or a defibrillator
Hemodynamically stable arrhythmia
Sustained ventricular tachycardia or cardiac arrest
*Contraindications*
Unstable angina pectoris
Heart failure (NYHA IV)
Acute endomyocarditis or other acute infections
Recent pulmonary artery embolism or phlebothrombosis
Hemodynamically relevant arrhythmia
Critical obstructions of the left ventricular outflow tract
Patients that cannot be rehabilitated because of physical, psychological or mental limitations

*NSTEMI* non-STEMI, *NYHA* New York Heart Association, *PCI* percutaneous coronary intervention, *SCORE* Systematic Coronary Risk Evaluation, *PROCAM* Prospective Cardiovascular Münster Study, *STEMI* ST segment elevation myocardial infarction

Currently, health insurances and the pension fund provide CR for their patients on a voluntary basis which is thus not mandated by law. As a result only a small minority of patients have access despite the fact that they have an otherwise qualifying indication. Even though scientific data document that patients benefit most if CR is initiated without a long wait [7], on average 36 days elapse before CR is started in Austria (Tab. 6 in [8]). Demand clearly exceeds supply, which could be easily overcome if insurance companies were willing to cooperate with a greater number outpatient CR facilities. If there were at least one in every town and/or valley, this would lead to a shorter wait and increased enrollment. Since most patients do not find facilities in their vicinity, it is not a surprise that <30% of eligible patients receive phase II and <20% phase III rehabilitation [9]. As a matter of fact, most Austrian cities do not even have a single outpatient CR facility, making it impossible for the vast majority of patients to continue with phase III, provided they ever enrolled in phase II.

In Austria, the health care system is traditionally inpatient orientated. Indeed, Austria still has the highest number of discharges per 100 inhabitants in Europe [10, 11]. This is also mirrored by CR, where of the 24 phase II and phase III rehabilitation facilities, 13 provide inpatient and 11 outpatient CR. Even though, at first glance, the inpatient:outpatient ratio seems well balanced, the number of discharged patients clearly demonstrates a striking difference between the two: of the 17,000 patients that undergo CR annually, 94% are in- and only 6% outpatients (Tab. 5a in [8]). This stands in stark contrast to the majority of other European countries, where not in- but outpatient CR is standard. Also, in Austria the percentage of patients that eventually participate in CR is lower than in many other European countries [9]. Again, too few outpatient CR facilities certainly contribute to this shortcoming.

Regardless of whether CR centers are private or public, all offer multidisciplinary CR and staff include all or some of the following: cardiologists, internal medicine specialists, sports medicine specialists, nurses, physiotherapists, occupational therapists, sport scientists, psychologists, dieticians, and others (for detail see [3]) All but three outpatient centers (2 owned by Pensionsversicherungsanstalt PVA; 1 private one) are accredited by the Austrian Working Group on Outpatient Cardiac Rehabilitation (AGAKAR), which establishes and assesses quality standards that are compulsory. These standards have previously been published in cooperation with the Austrian Society of Cardiology [3].

Although the incidence of myocardial infarction slightly decreased over the last ten years, cardiovascular diseases are still the leading cause of death (Fig. 1, [10]). Cardiovascular risk factors are well known; however, patients’ adherence to a heart-healthy lifestyle is suboptimal. It is the aim of both in- and outpatient CR to empower patients to combat modifiable cardiac risk factors. In order to do so, knowledge has to be expanded but also a network of health care professionals has to be established at the place of living, which is one of the main reasons why outpatient CR is so successful, be it during phase II or III.

**Fig. 1:**
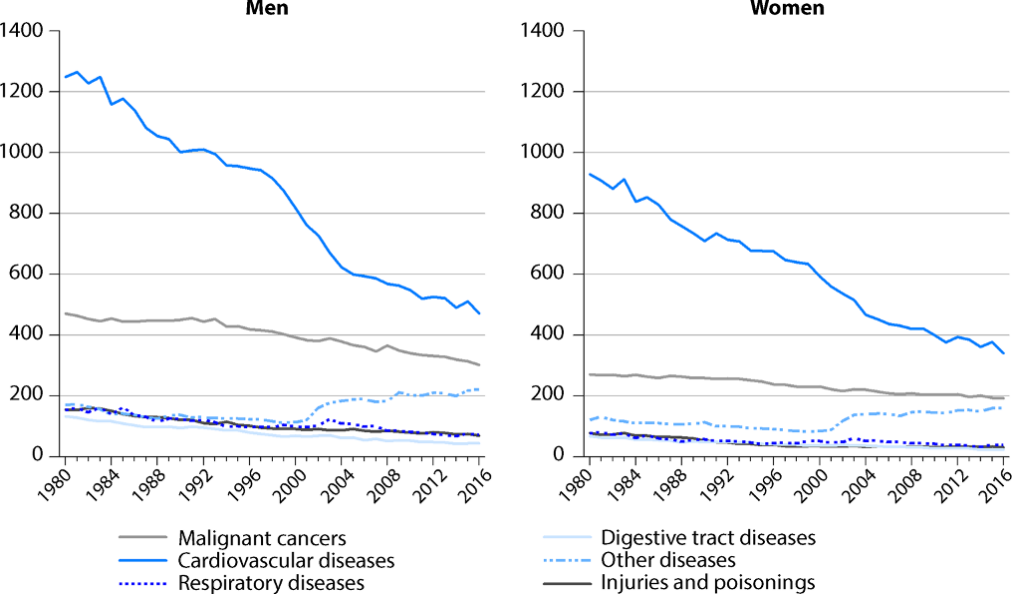
Age-adjusted mortality rate per 100,000 persons between 1980 and 2016 [10]

**Fig. 2:**
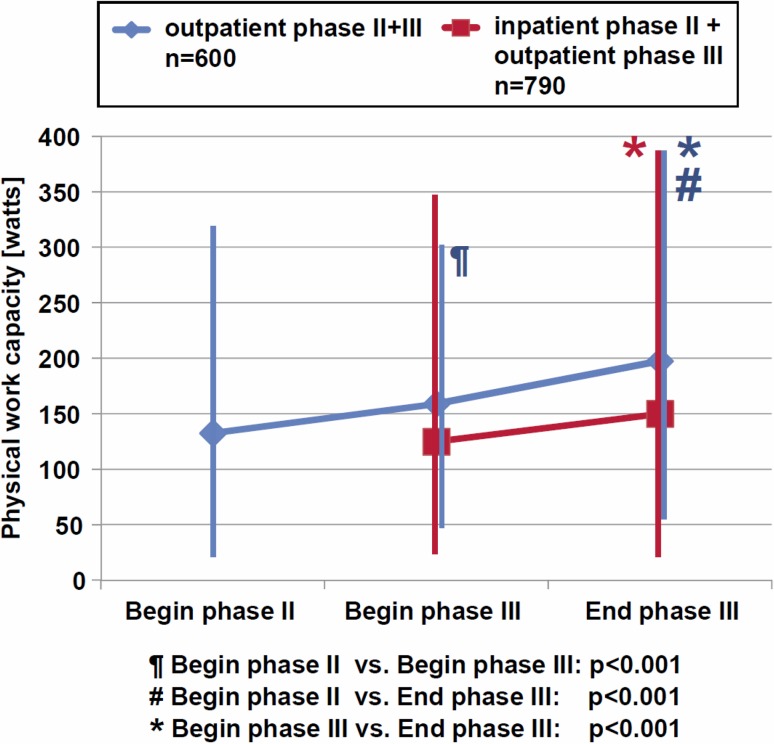
Physical work capacity during outpatient cardiac rehabilitation (*CR*) phase II and III [12]

Data documenting the success of inpatient CR are scarce. In fact, the PIN study demonstrated striking benefits during inpatient CR. Devastating however, were the one-year results, which were often as bad as or even worse than baseline. In contrast, the Austrian Working Group on Outpatient Cardiac Rehabilitation (AGAKAR) has published registry data from 2009–2011, where most cardiac risk factors improved strikingly after phase II and remained or even further improved after phase III rehabilitation (Fig. 2; [12]).

Uncounted studies as well as clinical experience have repeatedly shown that patients will not alter their behavior merely because of recommendations or gain in knowledge. In order to change behavior, lifelong interventions are necessary and a phase III program ideally followed by a lifelong phase IV program is the only viable solution. Exercise training, for example, is a therapeutic option which is very effective provided patients train on a regular basis. This is not different from any other treatment option, which can only exert its effects if it is being applied. Just like medication, the right form of exercise has to be chosen, has then to be titrated, and patient compliance has to be checked on a regular basis. Also, monitoring of effects and side effects is paramount and thus patients have to be followed-up lifelong. Exercise training is no different from smoking cessation or the adaption of a heart-healthy diet. Success of these effective preventive strategies depends to a large extent on an effective and adequate infrastructure that provides regular follow-up. As long as we pretend that patients are able to successfully battle modifiable risk factors themselves, we will be penalized with sobering yet devastating results. A look at the risk factor profile of average Austrians confirms this notion: whereas every person should exercise at least three times a week, less than half of the population (49%) exercised at least once a week, and only a third of all men and not even one quarter of all women exercised as advised. On a side note and to make matters worse, physical inactivity was reported by 18% of women and 22% of men [11].

Even though the number of smokers has continuously decreased over the last few decades, compared with other European countries Austrian numbers are still too high. Indeed, 23.3% of the population smoke regularly [13], which exceeds the EU-15-average of 22.1%. Compared to other EU countries, Austria has one of the highest percentages of young smokers [11]. Smoking at least once a week at the age of 15 was reported in 29% of girls and 25% of boys [14].

As the typical diet of the Austrian population contains too much fat, especially saturated fatty acids, 47.7% of the Austrian adult population is overweight, 12.4% of these even obese [11].

Diabetes mellitus, a key risk factor for cardiovascular diseases, was reported in 6% of the population [13].

If a “healthy” population is unable to perform long overdue lifestyle changes, how can one expect patients to do this long-term and without adequate help or infrastructure?!

## Conclusion

CR is effective both as in- and outpatient CR. Regardless of how CR was performed during phase II, patients benefitted from phase III, which is currently only available for too few patients. Efforts have to be increased in order to also make health insurance companies pay for CR, which shortly thereafter would lead to an adequate infrastructure, i. e., prevention centers in all cities and even remote valleys that offer CR in order to empower patients to successfully perform lifelong lifestyle changes. Ignoring the current and unsatisfactory situation will cause unnecessary harm to our patients and will subsequently lead to unaffordable health care costs for all.
